# Thyroid Hormones, Peripheral White Blood Count, and Dose of Basal Insulin Are Associated with Changes in Nerve Conduction Studies in Adolescents with Type 1 Diabetes

**DOI:** 10.3390/metabo11110795

**Published:** 2021-11-22

**Authors:** Marta Wysocka-Mincewicz, Marta Baszyńska-Wilk, Maria Mazur, Aleksandra Byczyńska, Monika Nowacka-Gotowiec

**Affiliations:** 1 Clinic of Endocrinology and Diabetology, Children’s Memorial Health Institute, 04-730 Warsaw, Poland; mbasz@wp.pl (M.B.-W.); m.mazur@ipczd.pl (M.M.); a.byczynska@ipczd.pl (A.B.); 2 Electromyography and Evoked Potential Laboratory, Children’s Memorial Health Institute, 04-730 Warsaw, Poland; m.nowacka-gotowiec@ipczd.pl

**Keywords:** type 1 diabetes, neuropathy, children, thyroid hormones, white blood count, risk factors

## Abstract

Type 1 diabetes (T1D) in the child population is the third most common chronic disease. Diabetic peripheral neuropathy (DPN) is a very disabling and silently developing complication. This prospective, observational study enrolled 182 (93 girls) patients with T1D, aged 16.5–18 years. The aim of the study was to assess the correlation between factors of diabetes metabolic control, blood count, thyroid hormones, thyroid-stimulating hormone (TSH), level of cortisol, vitamin D3, metabolic factors, demographic data, and nerve conduction study (NCS) parameters. We revealed that in multivariate regression models for almost all NCS parameters, beside height and diabetes duration, significant factors were basal insulin dose per kilogram of weight (BID/kg), body mass index (BMI), and thyroid hormones. For conduction velocities of the motor nerves, mean HbA1c exists in models. In all models for all NCS parameters there exists at least one parameter of peripheral white blood cell counts (predominantly monocytes). There is a significant influence of thyroid hormones, peripheral blood white cells count, and BID per weight on parameters of NCS. It is essential to take care of the proper insulin dose per weight of patients and the adequate proportion of basal to prandial insulin.

## 1. Introduction

Type 1 diabetes (T1D) in the child population is the third most common chronic disease, and its incidence is still increasing. Diabetes is burdened by the risk of micro and macrovascular complications [1]. The International Diabetes Federation estimates that 425 million people worldwide have diabetes [2], making it the largest global epidemic of the 21st century [3]. In total, 115 million people in China, 73 million in India, and 30 million in the United States have diabetes [4]. About USD 727 billion is directed towards diabetes and its complications, and this number persistently increases at an unsustainable rate [2]. Diabetic peripheral neuropathy (DPN) is a very disabling and silently developing complication. The lack of effective treatment for DPN highlights the importance of early diagnosis to prevent progression. DPN is the presence of symptoms and/or signs of peripheral nerve dysfunction caused by diabetes. The diagnosis is made after excluding other causes [5]. The results of studies on the prevalence of DPN in children vary widely. The range is from 9% to 97% of the diabetic population [6]. During the early stages, DPN is often asymptomatic; however, once symptoms and overt deficits have developed, it cannot be reversed. Unlike adults, children rarely develop symptoms of DPN; however, consequences of DPN are foot ulceration which can lead to amputation or chronic pain. Therefore, early prevention and detection are crucial to curb the progression of DPN in children.

Nerve conduction studies (NCS) are considered to be the gold standard for the diagnosis of DPN. The Toronto consensus [7] recommended using abnormal NCS with a symptom or sign to diagnose DPN. NCSs are non-invasive, standardized, and objective tests for measuring the dysfunction of large myelinated sensory and motor nerve fibres. The typical electrophysiological findings in DPN are amplitude reduction of the compound muscle action potential, slowing of sensory and motor nerve conduction velocity, and prolonged F-wave latency. Demyelinating neuropathy is diagnosed when there is a prolongation in latency and slowing in conduction velocity greater than 40% of the normal mean, while axonal degeneration can be determined by reduction in amplitudes of sensory or motor compound action potential. As previously observed, in the pediatric population, firstly, nerve conduction abnormalities were most pronounced in motor nerves of the leg (tibial and peroneal motor conduction velocity), followed, in the order of severity, by sensory nerves of the leg (sural sensory conduction velocity), sensory nerves of the arm (ulnar sensory conduction velocity), and motor nerves of the arm (ulnar motor conduction velocity) [8,9,10].

Major risk factors of DPN include diabetes duration, hyperglycemia, and age, height, followed by prediabetes, hypertension, dyslipidemia, and obesity [11]. The other significant factors detected in adult populations are smoking, insulin resistance, and hypoinsulinemia. The most important for health care providers are modifiable ones, such as hyperglycemia, hypertension, dyslipidemia, obesity, and smoking, because improving these results could prevent or adjourn complications. Hyperglycemia (commonly measured using glycated hemoglobin—HbA1c) has been the easiest and the most obvious target for intervention in preventing complications of diabetes. The Diabetes Control and Complications Trial (DCCT) [12] demonstrated that intensive glycemic control in type 1 diabetes monitored by HbA1c reduced the onset and progression of diabetic neuropathy. A new aspect of metabolic control is glucose variability. Christensen et al. revealed that greater coefficient of variation (CV) was associated with a decrease in incidents of symmetric abnormalities in sural nerve amplitude potential (SNAP), sural nerve conduction (composite measure of bilateral abnormalities in amplitude and/or velocity, SNC), and definite cardiovascular autonomic neuropathy (CAN) [13].

The other crucial metabolic risk factors are insulin resistance, hypertension, and obesity. Population-based studies from many countries indicated that obesity is common in patients with DPN [14,15,16,17]. The number of metabolic syndrome components, such as hypertriglyceridemia, hypertension, abdominal obesity, and low high-density lipoprotein (HDL) levels, is consistently associated with diabetic neuropathy in patients with T2DM [14,15] and selected T1DM cohorts [18], independently of glycemic control.

As mentioned above, factors set for the risk of diabetic neuropathy are still not established, especially in the pediatric population. As a result, the aim of the study was to analyze all recorded parameters, including demographical, vital, biochemical, metabolic, hematological, and endocrinological measurements, as well as treatment data.

## 2. Results

The results of multiple regression models are presented in Table 1 and Table 2. We revealed that in models, there are correlations between almost all nerve conduction parameters and demographic factors like height and diabetes duration, basal daily dose per kilogram of weight, BMI, and thyroid hormone. For conduction velocities of the motor nerves, mean HbA1c exists in models. In all models for all NCS parameters at least one parameter of white blood cell counts exists (predominantly monocytes, but also eosinophils and neutrophils to lymphocytes index).

Comparing our results differently, when we looked at the motor and sensory nerves concurrently, we detected that for conduction velocity, the main factors were vital signs (height, weight, BMI), BID, and duration of CSII. In comparing the potential amplitude of sensory and motor nerves, there were significant height, thyroid hormones, diabetes duration, and peripheral white cell count parameters, especially monocytes. Nevertheless, when we compare the risk factors set for sensory and motor latency, repetitive parameters were weight/BMI, BID/kg or TDI/kg, peripheral blood monocytes count, and neutrophils or NLR.

## 3. Discussion

Knowledge of risk factors of such harming and disabling morbidity as diabetic neuropathy allow to build the prevention programs and delay this complication. This is the first study when so many parameters that could influence NCS have been analyzed in such a large population of youths. In this study, we showed risk factor sets for all NCS parameters in a group of patients with T1D without any late complications, with very similar age, without disturbances in laboratory tests. As proven by EURODIAB the age, diabetes duration, and poor metabolic control are the major risk factors for developing DPN [19]. The independent influence of diabetes duration and long-term poor metabolic control on the risk of diabetic retinopathy and nephropathy confirmed the Linkoping Diabetes Complications Study results on young adults with T1D [20]. We could not test the influence of age because of our study group’s very narrow range of age. Results of our study are compatible with previous studies that weight, height, BMI, and BMI Z-score are significant vital risk factors for peripheral nerves damage [21,22]. Height plays a role in the pathogenesis of DPN because of the length-dependent pattern of the disease as a measure of nerve fiber length. Such association was also observed in the EURODIAB IDDM Complications Study [21]. Show et al. revealed that height was a significant (*p* < 0.001) independent risk factor of DPN, increasing its prevalence by 36% for every 5 cm increment (odds ratio (OR): 1.36, 95% confidence interval (CI): 1.19–1.57) [23].

Callaghan et al. in a multivariable logistic regression model investigating the metabolic syndrome components revealed that hyperglycemia (odds ratio (OR) 2.60, 95% CI 1.77–3.80) and weight (OR 1.09, 95% CI 1.02–1.18) were significantly associated with the peripheral neuropathy [14], similarly to German [10] and our results.

In the meta-analysis of risk factors for DPN in T2D patients, Liu et al. revealed that diabetes duration (mean differences MD 2.5, 95%CI 1.71–3.29), age (MD 4,00, 95%CI 3.05–4.95), HbA1c (MD 0.48, 95%CI 0.33–0.64), and diabetic retinopathy are associated with significantly increased risks of DPN, but BMI, smoking, total triglyceride, and total cholesterol did not increase risk [24]. Increased glucose levels lead to glucose metabolism via the polyol and hexosamine pathways, resulting in increased reactive oxygen species and inflammation, mainly owing to mitochondrial injury [25], that when dysfunctional produces low energy and loses the ability to normally traffic down axons, further promoting axonal disruption and injury, which leads to nervous system dysfunction [26]. Increased glucose levels lead to the glycation of numerous structural and functional proteins to produce advanced glycation end-products (AGEs), which interact via the AGE-specific receptor (RAGE), modify gene expression and intracellular signaling, and promote the release of pro-inflammatory molecules and free radicals [27]. Additionally, excessive free fatty acids catabolized by β-oxidation in response to hyperlipidemia can injure the peripheral nervous system, particularly Schwann cells [28], through reactive oxygen species generation and systemic and local inflammation via macrophage activation.

Strict glycemic control is an undeniable factor for the prevention of DPN in T1D patients [29,30]. It was calculated that in hyperglycemia, each percent of increasing HbA1c frequency of DPN increases 10-15% [31]. It was very interesting that diabetes duration and mean HbA1c exist in our study models, but not in all. Diabetes duration was in all nerve models of potential amplitude, but HbA1c was in conduction velocity models and rather in nerves of the lower extremity, which is comparable with results of DCCT [32]. It is possible that patients have significantly better regeneration at a young age, which can diminish harming T1D impact and could suggest plasticity of the nervous system at a young age. The second possibility is that this is the first generation of young persons using all diabetes duration insulin pumps, and many of them use continuous glucose monitoring systems (CGMS), which helps decrease glycemic variations. There is a lack of evidence studies that proved the strong impact of large glycemic daily variability on the nervous system, because it needs CGMS for a long period, and a project of long-term studies. There is a lack of glycemic variation parameters showing a very long time of observations, which is necessary for persistent nerve fiber damage. Our assumption confirms results of the Ziegler et al. study, which after 24-year prospective observation strictly (near-normoglycemic) controlled patients with T1D observed from diagnosis do not develop DPN [30].

As in the previous studies, current metabolic control was not an important factor in our analysis, but only a long time of poor glycemic control influenced the nerve fiber [33]. In our study, diabetes duration and duration of using CSII were important factors for many of NCS parameters. Similarly to a meta-analysis performed by Liu et al. [24], in our models lipid parameters did not exist as significant factors for changes in nerve function, but it could be due to the fact of normal values of lipids in our population.

The most important result of our study seems to be the great impact of basal insulin dose per kilogram of weight (BID/kg), which shows the influence of persistent higher levels of insulinemia in the body. In T1D, it is very difficult to recognize the insulin resistance. Adolescents and adults with T1D have reduced insulin sensitivity, which is linked to initiation and progression of micro- and macrovascular complications [34]. Due to the nature of this type of diabetes, oral and intravenous glucose tolerance tests are prohibited, and the gold standard in diagnosing insulin sensitivity remains the hyperinsulinemic-euglycemic clamp, which is difficult and time-consuming. The eIS-CACti model of insulin resistance includes waist circumference, TDI/per kg body weight, TG, and diastolic blood pressure [34]. This model explains 63% of the variance in the glucose disposal rate in clump studies. Interestingly, in none of our analyses was TDI important, nor percent of basal insulin, and TDI/kg existed only twice (only in the parameter of distal latency of nerves lower extremity). In the study of Lee et al. [35], in the group of 86 patients with T2D, it was identified that insulin resistance is a major independent risk factor of DPN. The authors in the prospective part of the study revealed that initial insulin resistance was positively associated with impairment of sural sensory nerve action potential after 6 years of observation (r = 0.6, *p* = 0.001), with adjustment for age, gender, and height [36]. Higher basal insulin dose could be compared to conventional insulin therapy which was revealed in DCCT as more harmful than the intensive one [32], because of the metabolism in an all-time higher concentration of insulin. In our study, BID/kg was important in all conduction parameters of sensory nerves. Interestingly, the total daily dose per kilogram (TDI/kg) was important only in models for latency of nerves in lower extremities, which could suggest a significant influence on long nerve fibers.

Thyroid hormones are the most important for human metabolism, growth, and nervous system function and development. There is a lack of studies on the influence of thyroid status on complications in Type 1 diabetic children, but Falkowski et al. [37] in an adult population showed that higher FT3 (but not TSH and FT4) is associated with lower prevalence of microangiopathic complications assessed as the presence of at least one of the measured parameters (direct ophthalmoscopy, urinary albumin excretion in 24 h urine collection, peripheral neuropathy, and cardiovascular autonomic neuropathy). Our study analyzed the influence of thyroid hormones in the population without clinical signs of microangiopathy, not as Falkowski et al. much later, when patients have clinically visible microvascular complications. In the Chinese population of 605 patients with T2D, the level of TSH was significantly and independently associated with DPN (evaluated by signs, symptoms, and electromyograms) [38]. Levels of FT3 and FT4 in our significant models were present for distal latencies and potential amplitudes of motor as well as sensory nerves, of upper and lower extremities. Additionally, Zhu and Yang observed that FT3 serum level was associated with results of conduction velocities and potential amplitude in conduction studies of patients with T2D [39]. Lower serum levels of thyroid hormones accelerate the endothelial dysfunction, which contributes to microangiopathy partially causing the DPN [40].

The next interesting result of our study was the influence of peripheral leukocytes (white blood cells) count, especially monocytes, and neutrophils/lymphocytes ratio. These easy and cheap markers of inflammation have been shown as independent risk factors for diabetic retinopathy, maculopathy, and diabetic kidney disease. Those parameters are associated with insulin resistance, the onset of type 2 diabetes [41,42,43,44], coronary artery disease (CAD) [45], and stroke [46,47] and diabetes micro- and macrovascular complications [47,48,49,50,51]. Young et al. in the study of 1073 patients with T2D showed that peripheral monocyte count was an independent predictor of all-cause mortality, especially in the case of coexistence of macrovascular complications [52]. Peripheral leukocytes consist of polymorphonuclear cells, including monocytes as well as lymphocytes. Polymorpho- and mononuclear leukocytes can be activated by advanced glycation end products, oxidative stress, angiotensin II, and cytokines in a state of hyperglycemia [49]. In all our models one or more peripheral white blood count parameters exist, which suggest involvement of inflammation in the progress of T1D complications. In the Vural and Gümüsyayla nerve conduction study of 90 patients with DPN, 92 diabetic patients without DPN and 67 healthy controls, the authors observed that monocytes count was not different, but the neutrophil count was higher in a group with DPN [53]. The lymphocytes count of patients with non-DPN was significantly lower than in the other groups. Multivariate logistic regression analysis demonstrated that age, monocyte/cholesterol fraction HDL ratio (MHR), neutrophil, HbA1c, and diabetes duration were independent predictors of DPN. In this study, potential amplitude of the posterior tibial nerve was negatively correlated with monocytes counts and MHR. In our study, monocytes count was associated with changes in all parameters of the tibial posterior nerve and both sensory potential amplitudes (median and sural nerves). The previous studies demonstrated that the neutrophil/lymphocyte ratio (NLR), as an indicator of systemic inflammation, increases in patients with diabetes [54], in diabetic retinopathy and nephropathy, and in diabetic patients with coronary artery disease. In a recent study, Liu et al. observed that patients with high NLR exhibited a lower nerve conduction velocity and that such patients were more likely to develop polyneuropathy [55]. Similarly, in our study, this parameter existed in models for conduction velocities of median motor and sural nerves and distal latency of median motor nerve.

Lower hemoglobin concentration, the main protein carrying oxygen, is associated with ischemia of retina [56], and diabetic nephropathy [57]. In our study, this parameter was significant only in one model, for motor conduction velocity of the tibial posterior nerve, but in models of both tested motor nerves, MCHC has a statistically significant influence. This result could mean that level of oxygenation is more significant for the long motor nerve function, or only firstly visible in these prone to injury ones.

Niu et al. confirmed that a low vitamin D level is associated with DPN in diabetic patients over 65 years of age and might be a useful predictor of DPN. The authors showed that vitamin D level was not associated with DPN in young and middle-aged patients [58]. In our analysis, the level of vitamin D did not exist in any of the models.

This is the first, to our knowledge, such in-depth analysis of many potential risk factors of diabetic neuropathy in the population of adolescents with T1D. This is the first time when impact on nerves function of thyroid hormones levels, peripheral white cell counts, total hemoglobin/MCHC, and duration of using CSII were revealed. In this study, the amount of basal insulin per weight was pronounced for the first time and was shown as a more important factor than total daily insulin dose. However, some limitations of the study have been noticed. We have a population without any clinical signs and symptoms of DPN, because of that we could not prepare a model of logistic regression and more precisely showed the risk factors for the whole peripheral nervous system, not for a particular parameter. It is very inconvenient that we did not analyze even more parameters like mean platelet volume (MPV), which assesses level of platelet activation, or monocytes to HDL cholesterol ratio which could pronounce epithelial dysfunction.

## 4. Materials and Methods

It was a prospective, observational study in a group of adolescents diagnosed with T1D based on International Society for Pediatric and Adolescent Diabetes (ISPAD) criteria, with insulin treatment. Inclusion criteria were previously recognized T1D, not in remission status, no DPN, and assent agreement for participation. Patients remain under the control of the Clinic of Endocrinology and Diabetology of Children’s Memorial Health Institute. The study was approved by the Bioethics Committee Children’s Memorial Health Institute in Warsaw and followed the tenets of the Declaration of Helsinki no 24/KBE/2020. A written informed consent was obtained from the patient’s legal guardians and from patients after explaining the nature of the non-invasive study before the tests started.

A total of 182 youths participated in the study (93 girls, 89 boys), demographic, biochemical, and treatment data in Table 3. All adolescents in the study were Caucasian. Neurological assessment was proposed to adolescents before transmission to clinics for adults. Conduction studies of the median, tibial, and sural nerves were performed on patients.

A modified neuropathy disability score was used to diagnose DPN [59,60]. The modified neuropathy disability score (NDS) was derived for neurological examination of vibration perception, sharp/blunt sensation, temperature sensation using the Neurotip device, and ankle reflexes using a reflex hammer. A score of 0 was given for normal response and 1 for an abnormal response for each individual test component, independently for each leg (total 10 points). A modified NDS ≥ 3 indicates neuropathy, with higher scores indicating more severe disease [60]. Study group patients with NDS < 3 were included (all patients have NDS = 0). No patient had symptoms and signs of DPN. None of the patients were affected by system hypertension, renal dysfunction, nor diabetic retinopathy.

The electrophysiological tests were done for all patients by the Sierra Summit electromyography machine (Cadwell) which permits the storage of 16 F responses and the automatic calculation of the parameters. NCS was performed with conventional neurophysiological techniques using surface electrodes. Patients were placed in a supine position in relative relaxation, in-room temperature between 22 and 24 °C. Extremities were warmed up to a surface temperature between 32 and 36 °C. The simplified NCS protocol for non-symptomatic patients was used. For each patient, the standard nerve conduction measurements were performed on tibial motor and sural sensory nerves in the right lower extremity, and median nerve (motor and sensory) in the right upper extremity. Motor nerve conduction velocity (MCV), compound muscle action potential (CMAP), distal motor latency (DML) were determined in the motor nerves. Sensory nerve conduction velocity (SCV), sensory nerve action potential amplitude (SNAP), and distal sensory latency (DSL) were determined in the sensory nerves (median—orthodromic stimulation; sural—antidromic stimulation). The electrophysiological studies of all patients were performed by the same physician.

We assessed factors of diabetes metabolic control: glycated hemoglobin A1c (HbA1c) current to the study, and mean value for whole diabetes duration (minimum 4 tests per each year of diabetes duration), total daily insulin dose per kilogram of weight (TDI/kg), mean total daily insulin (taken from pump memory or profile led on a hospital, TDI), basal insulin dose (BID), percent of basal insulin (percent of basal from TDI), basal insulin per kilogram of weight (BID/kg).

The group treated using multiple dose insulin (MDI group) included 29 children (mean diabetes duration 6.1 ± 4.45 years) treated by pens in scheme basal-bolus-functionally, meaning dose of insulin analogue is accounted for depending on the amount of food and correction, or children with stiff doses for main meals. The group treated using continuous subcutaneous insulin infusion (CSII group) consisted of 153 children treated with an insulin pump for more than a year (mean duration of CSII 6.1 ± 3.5 years, mean duration of diabetes 7.85 ± 4.1 years). However, not all the patients strictly followed the recommendations of the diabetes care team (stiff doses for meals, not weighted meals).

The other metabolic/biochemical factors assessed in the study (all samples were taken fasting): lipids (levels of total cholesterol, low-density lipoprotein (LDL), high-density lipoprotein (HDL), triglycerides (TG)), serum uric acid, plasma creatinine, current microalbuminuria (mg/L), transaminase alanine, total bilirubin, serum calcium level, and hormonal status: thyroid function (thyrotropin (TSH), free thyroxine (FT4), free triiodothyronine (FT3)), level of cortisol measured with the chemiluminescent immunoassay method on the Alinity Abbott analytical system (Lake Bluff, Illinois, USA); blood count (white blood count, neutrophils count, lymphocytes count, monocytes, eosinophils, total hemoglobin level, mean concentration of hemoglobin in a red cell)—analyzed by SYSMEX XN 1000 A (Norderstedt, Germany) and level of 25(OH)D3 vitamin measured with the chemiluminescent immunoassay method on the Immunodiagnostic Systems (IDS, Boldon UK).

The following demographic data was accounted for in the study: weight, height, body mass index (BMI), Z-score of weight, height and BMI, age, age at diabetes onset, diabetes duration, duration of continuous subcutaneous insulin infusion (CSII) by a pump.

BMI Z-score was calculated using the LMS method based on Box-Cox transformation [61,62]:

(1) $$z-score\left(x\right)=\frac{{\left(\frac{X}{M}\right)}^{L}-1}{LxS}$$

*X*—measured anthropometric parameter (ex. heigh, BMI); *M*—median of the value, *L*—power of Box-Cox’a transformacy; *S*—variability coefficient. *L*, *M*, and *S* values were taken from reference tables for a chosen anthropometric parameter for determining age and sex (were printed by Kułaga et al.) [63,64].

## 5. Statistical Analysis

The data was described by mean, median, standard deviation, and minimal and maximum values. For the analysis of correlations between parameters, multiple regression analysis was used. In the first step, all collected descriptive and metabolic data were taken to multiple regression analysis, then for the model statistically significant parameters were chosen. A level *p* < 0.05 was recognized as statistically significant. Tests were performed using TIBCO Software Inc. (2017) Statistica version 13.3 StatSoft Company (Lakeway, TX, USA).

## 6. Conclusions

Analysis of risk factors for changes in peripheral nerves should take into consideration long-standing and constants of parameters because short-acting, quickly changeable parameters could not have a persistent impact on function of the nerve cell. In a group of adolescents with type 1 diabetes, peripheral nerves’ function depends on more parameters than previously assessed. In our study, we showed a significant influence of thyroid hormones, peripheral blood white cells count, and basal insulin dose per weight on parameters of peripheral nerves conduction studies. It is essential to take care of the proper insulin dose per weight of patients and the adequate proportion of basal to prandial insulin. Current metabolic control was not an important factor for conduction studies, only the mean value of glycated hemoglobin was associated with changes. There is a need for long-term observation in prospective studies that will assess the influence of thyroid hormones, peripheral blood counts, metabolic syndrome, and therapy methods on the function of peripheral nerves.

## Figures and Tables

**Table 1:** Multiple regression models for nerve conduction study parameters nerves.

	Median Motor Nerve	Tibial Posterior Nerve
Motor conduction velocity (MCV)	R = 0.38, R2 = 0.15, *p* < 0.0005 Height ß = 0.25, *p* = 0.07 Weight ß = −0.34, *p* < 0.02 BMI Z-score ß = −2.95, *p* < 0.05 BID/kg ß = −9.37, *p* = 0.09 HbA1c mean ß = −1.05, *p* < 0.001 Duration of CSII ß = −0.49, *p* < 0.001 NLR ß = −0.31, *p* = 0.5	R = 0.56, R2 = 0.31, *p* < 0.00000 Height ß = −0.023, *p* < 0.0001 Total Hb ß = 1.27, *p* < 0.01 MCHC ß = −1.94, *p* < 0.0001 BID/kg ß = −11.17, *p* < 0.05 HbA1c mean ß = −0.7, *p* = 0.1 Monocytes ß = −1.38, *p* = 0.6
Distal motor latency (DML)	R = 0.39, R2 = 0.15, *p* < 0.001 Height ß = 0.09, *p* < 0.005 Weight ß = −0.1, *p* < 0.02 BMI ß = −0.33, *p* < 0.001 BID/kg ß = −1.03, *p* < 0.05 WBC ß = −0.15, *p* < 0.05 Duration of CSII ß = −0.12, *p* = 0.4 NLR ß = −0.22, *p* = 0.15	R = 0.36, R2 = 0.13, *p* < 0.0005 HbA1c mean ß = 0.11, *p* < 0.003 Weight ß = 0.021, *p* < 0.005 BMI ß = −0.8, *p* < 0.005 TDI/kg ß = −0.11, *p* = 0.07 Monocytes ß = −0.6, *p* < 0.005 FT4 ß = 1.65, *p* < 0.01
Compound muscle action potential (CMAP)	R = 0.41, R2 = 0.16, *p* < 0.02 Height ß = 0.23, *p* < 0.05 Uric Acid ß = −1.7, *p* < 0.01 MCHC ß = −1.09, *p* < 0.02 FT4 ß = −7.75, *p* < 0.05 Eosinophiles ß = 5.93, *p* < 0.04 Calcium total ß = 7.6, *p* = 0.2 Diabetes duration ß = 3.9, *p* = 0.2 FT3 ß = −1.9, *p* = 0.02	R = 0.48, R2 = 0.23, *p* < 0.00000 Weight z-score ß = −6.21, *p* < 0.0001 HbA1c mean ß = −0.9, *p* < 0.02 MCHC ß = −0.92, *p* < 0.04 FT3 ß = −3.4, *p* < 0.02 Monocytes ß = −2.8, *p* < 0.04 Creatinine ß = 6.6, *p* < 0.02 Microalbuminuria ß = 0.3, *p* < 0.005 Diabetes duration ß = 3.9, *p* = 0.2

BMI—body mass index; HbA1c—glycated hemoglobin; TDI—total daily insulin; BID—basal insulin dose; CSII—continuous subcutaneous insulin infusion; MCHC—mean corpuscular hemoglobin concentration; NLR—Neutrophil–Lymphocyte Ratio; TSH—thyroid-stimulating hormone; FT3—free triiodothyronine; FT4—free thyroxine.

**Table 2:** Multiple regression models for nerve conduction studies of sensory nerves.

	Median Sensory Nerve	Sural Nerve
Sensory nerve conduction velocity (SCV)	R = 0.47, R2 = 0.22, *p* < 0.00000 Height ß = −1.01, *p* < 0.0005 Weight ß = 1.17, *p* < 0.001 BMI ß = −3.25, *p* < 0.002 BID/kg ß = −10.3, *p* < 0.03 Eosinophiles ß = −4.15, *p* < 0.05 TSH ß = −0.92, *p* < 0.002 Duration of CSII ß = 0.18, *p* = 0.1	R = 0.39, R2 = 0.15, *p* < 0.001 Height ß = 0.09, *p* < 0.005 Weight ß = −0.1, *p* < 0.02 BMI ß = −0.33, *p* < 0.001 BID/kg ß = −1.03, *p* < 0.05 WBC ß = −0.15, *p* < 0.05 Duration of CSII ß = −0.12, *p* = 0.4 NLR ß = −0.22, *p* = 0.15
Distal sensory latency (DSL)	R = 0.57, R2 = 0.33, *p* < 0.00000 Height ß = 0.06, *p* < 0.002 Weight ß = −0.06, *p* < 0.005 BMI ß = 0.17, *p* < 0.005 BID/kg ß = 0.55, *p* < 0.05 Neutrophiles ß = −0.2, *p* < 0.05 TSH ß = 0.02, *p* < 0.05 Duration of CSII ß = −0.1, *p* = 0.2	R = 0.36, R2 = 0.13, *p* < 0.001 HbA1c mean ß = 0.07, *p* < 0.02 BMI z-score ß = −0.12, *p* < 0.02 TDI/kg ß = −1.28, *p* < 0.001 FT4 ß = 0.98, *p* < 0.01 FT3 ß = −0.07, *p* = 0.2 LDL cholesterol ß = −0.22, *p* = 0.08 Monocytes ß = −0.16, *p* = 0.2
Sensory nerve action potential amplitude (SNAP)	R = 0.52, R2 = 0.26, *p* < 0.00001 Height ß = −3.54, *p* < 0.01 BMI ß = −12.54, *p* < 0.005 BID/kg ß = −45.2, *p* < 0.05 Diabetes duration ß = 15.6, *p* < 0.05 Age at onset ß = 15.9, *p* < 0.05 Neutrophiles ß = 6.1, *p* < 0.02 PLT ß = 0.06, *p* < 0.03 FT4 ß = −19.8, *p* = 0.2 Monocytes ß = 8.3, *p* = 0.06	R = 0.4, R2 = 0.16, *p* < 0.0001 Height ß = −0.37, *p* < 0.0001 BMI ß = −0.89, *p* < 0.001 BID/kg ß = −11.8, *p* = 0.2 Monocytes ß = −3.7, *p* = 0.2 Calcium total ß = 11.2, *p* = 0.3 Diabetes duration ß = −0.3, *p* = 0.1

BMI—body mass index; HbA1c—glycated hemoglobin; TDI—total daily insulin; BID—basal insulin dose; CSII—continuous subcutaneous insulin infusion; MCHC—mean corpuscular hemoglobin concentration; WBC—white blood cell; PLT—platelet count; NLR—Neutrophil–Lymphocyte Ratio; LDL—low density lipoprotein; HDL—high density lipoprotein; ALAT—alanine aminotransferase; TSH—thyroid-stimulating hormone; FT3—free triiodothyronine; FT4—free thyroxine.

**Table 3:** Demographic, metabolic, biochemical, and treatment data of the studied group.

Parameter	Mean+/−SD	Median	Min–Max
Age (years)	17.93+/−0.2	18.0	16.5–18.0
Age at onset (years)	10.29+/−4.17	10.66	1.17–17.19
Weight (kg)	69.76+/−11.45	69.45	45.5–98
Height (cm)	173.78+/−9.57	173.9	154–200
BMI (kg/m^2^)	23.1+/−3.42	22.59	16.26–33.91
BMI Z-score	0.44+/−1.02	0.48	–2.42–2.67
Diabetes duration (years)	7.58+/−4.16	7.21	0.65–16.94
Current HbA1c (%)	8.07+/−1.74	7.8	5.2–15.6
Mean HbA1c (%)	7.94+/−1.37	7.71	5.1–13.34
TDI (u)	52.41+/−18.2	50.71	5.0–99.4
TDI/kg of weight (u/kg)	0.75+/−0.23	0.75	0.09–1.35
BID (u)	18.7+/−6.87	17.85	2.85–50
BID/kg of weight (u/kg)	0.27+/−0.09	0.26	0.04–0.61
% of basal insulin	36.45+/−8.62	36.17	8.81–60.0
Duration of CSII (years)	6.01+/−3.52	5.83	0.01–15.15
Hemoglobin (g/L)	14.55+/−1.5	14.6	5.7–17.4
MCHC (g/dL)	33.84+/−1.14	33.7	31–37.4
WBC (10^3/uL)	6.27+/−1.63	6.2	2–14
PLT (10^3/uL)	257.99+/−60.68	263	25–408
Neutrophil (10^3/uL)	3.31+/−1.39	3.02	1.17–11.4
Lymphocytes (10^3/uL)	2.18+/−0.59	2.18	0.64–4.05
Monocytes (10^3/uL)	0.57+/−0.35	0.54	0.12–4.3
Eosinophils (10^3/uL)	0.2+/−0.18	0.14	0.02–1.32
NLR	1.63+/−0.9	1.42	0.57–7.81
Cholesterol total (mg/dL)	166.17+/−33.56	160	98–309
LDL cholesterol (mg/dL)	88.02+/−27.77	84.8	36–187
HDL cholesterol (mg/dL)	60.3+/−13.5	59	33–99
Triglycerides (mg/dL)	91.99+/−61.9	78	30–615
Uric acid (mg/dL)	4.32+/−0.99	4.15	2.2–6.8
Microalbuminuria (mg/L)	13.6+/−38.74	6.95	3–500
Creatinine serum (mg/dL)	0.81+/−0.19	0.81	0.39–2.26
Cystatin (mg/L)	0.8+/−0.12	0.8	0.53–1.16
ALAT (U/L)	14.23+/−9.42	12.0	5.0–67.0
Bilirubin total (mg/dL)	0.84+/−0.47	0.72	0.16–2.91
25(OH)D3 vitamin (ng/mL)	24.22+/−9.48	23.3	7.0–76.6
Calcium total (mmol/L)	2.37+/−0.08	2.38	2.13–2.53
TSH (µU/mL)	2.05+/−1.2	1.82	0.49–10.75
FT3 (pg/mL)	2.93+/−0.48	2.97	0.81–4.02
FT4 (ng/dL)	0.94+/−0.11	0.94	0.63–1.24
Cortisol (µg/dL)	15.15+/−3.73	15.4	5.6–24.3
IgA (g/L)	1.86+/−0.74	1.76	0.1–4.5

SD—standard deviation; BMI—body mass index; HbA1c—glycated hemoglobin; TDI—total daily insulin; BID—basal insulin dose; CSII—continuous subcutaneous insulin infusion; MCHC—mean corpuscular hemoglobin concentration; WBC—white blood cell; PLT—platelet count; NLR—Neutrophil–Lymphocyte Ratio; LDL—low density lipoprotein; HDL—high density lipoprotein; ALAT—alanine aminotransferase; TSH—thyroid-stimulating hormone; FT3—free triiodothyronine; FT4—free thyroxine; IgA—immunoglobulin A.

## Data Availability

The data presented in this study are available on request from the corresponding author. The data are not publicly available due to the privacy of the patients who assisted in the research.
